# Supplementary material for: Complexome profiling of the Chlamydomonas psb28 mutant reveals TEF5 as an early PSII assembly factor
Source: Plant Cell. 2025 Mar 17;37(6):koaf055. doi: 10.1093/plcell/koaf055 (PMC12164590; doi:10.1093/plcell/koaf055)
Supplement: koaf055_Supplementary_Data [file koaf055_supplementary_data.zip › psb28_interactive.zip!/plots/intensity/qualityPlots/peptides/PDC2_Cre02.g099850.t1.1.html]

### Cre02.g099850.t1.1

Trivial name: PDC2  
SeqCoverage [median]: 24.90  
Mass: 51.70 kDa  
  
 

| Sequence | MissedCleavage | Mass [Da] | wtIntensity | muIntensity | Score |
| --- | --- | --- | --- | --- | --- |
| ATASTAGDQDPHIYK | 0 | 1573.74 | 3255.33 | 0.00 | 114.56 |
| ATASTAGDQDPHIYKK | 1 | 1701.83 | 391.60 | 0.00 | 63.71 |
| FADESPKPER | 1 | 1174.56 | 812.43 | 382.86 | 65.50 |
| FRGHSLADPDELR | 1 | 1511.75 | 542.85 | 0.00 | 39.42 |
| GFGIAEDGR | 0 | 920.44 | 0.00 | 492.31 | 55.53 |
| GQLLENVFADPR | 0 | 1357.70 | 2899.35 | 1788.38 | 107.24 |
| KGPAFGMPGVLVDGMDVLK | 1 | 1930.01 | 519.99 | 1159.32 | 56.41 |
| RGEGPTLIEAETYR | 1 | 1590.80 | 7016.26 | 8417.91 | 138.77 |
| SPIVTPETAK | 0 | 1041.57 | 2909.46 | 3177.41 | 98.03 |
| YMIDNGLASEADIK | 0 | 1538.73 | 0.00 | 830.82 | 81.92 |
|  |  |  | 18347.3 | 16249.0 |  |
